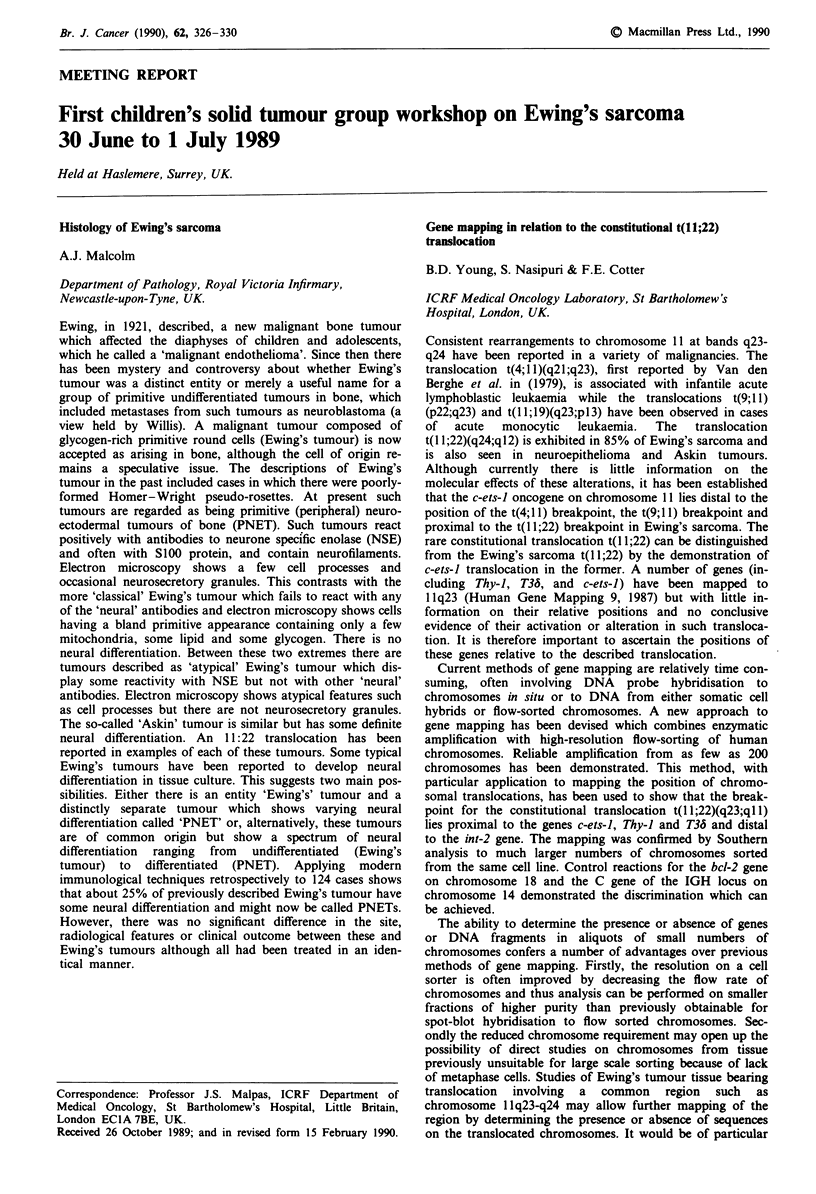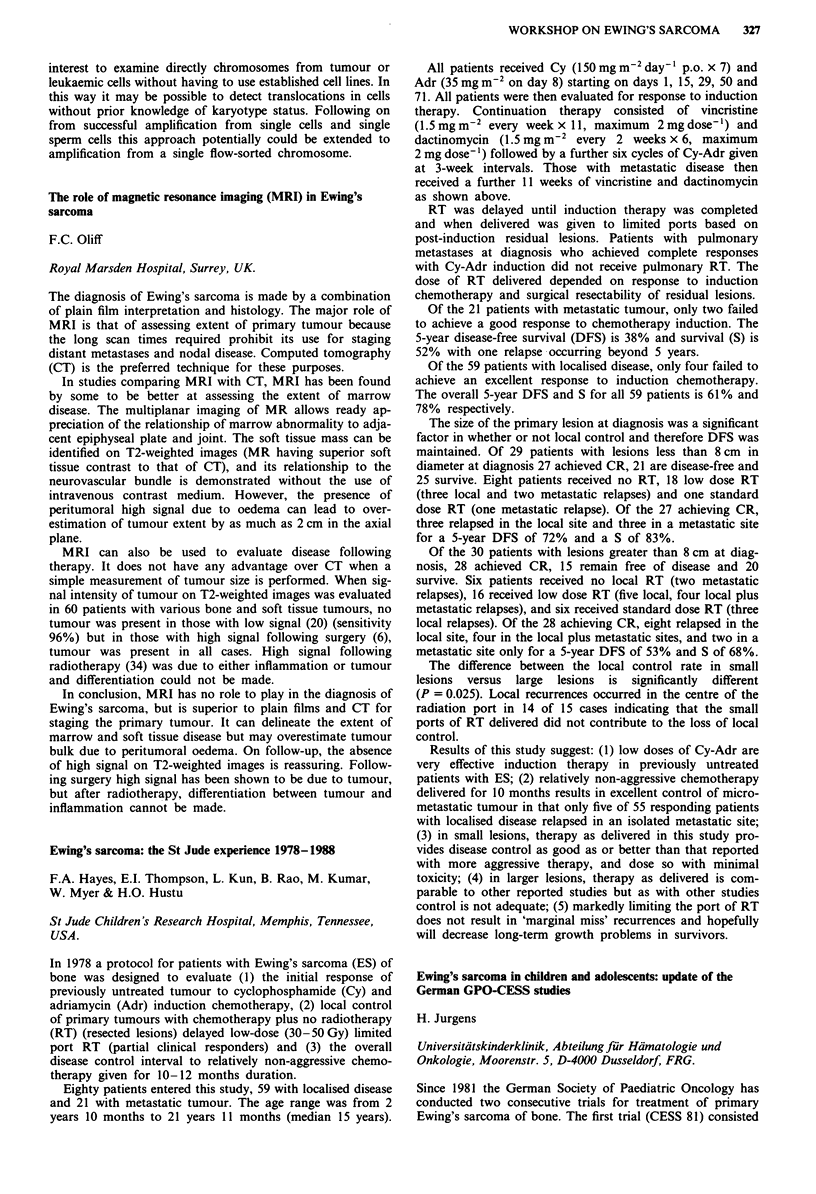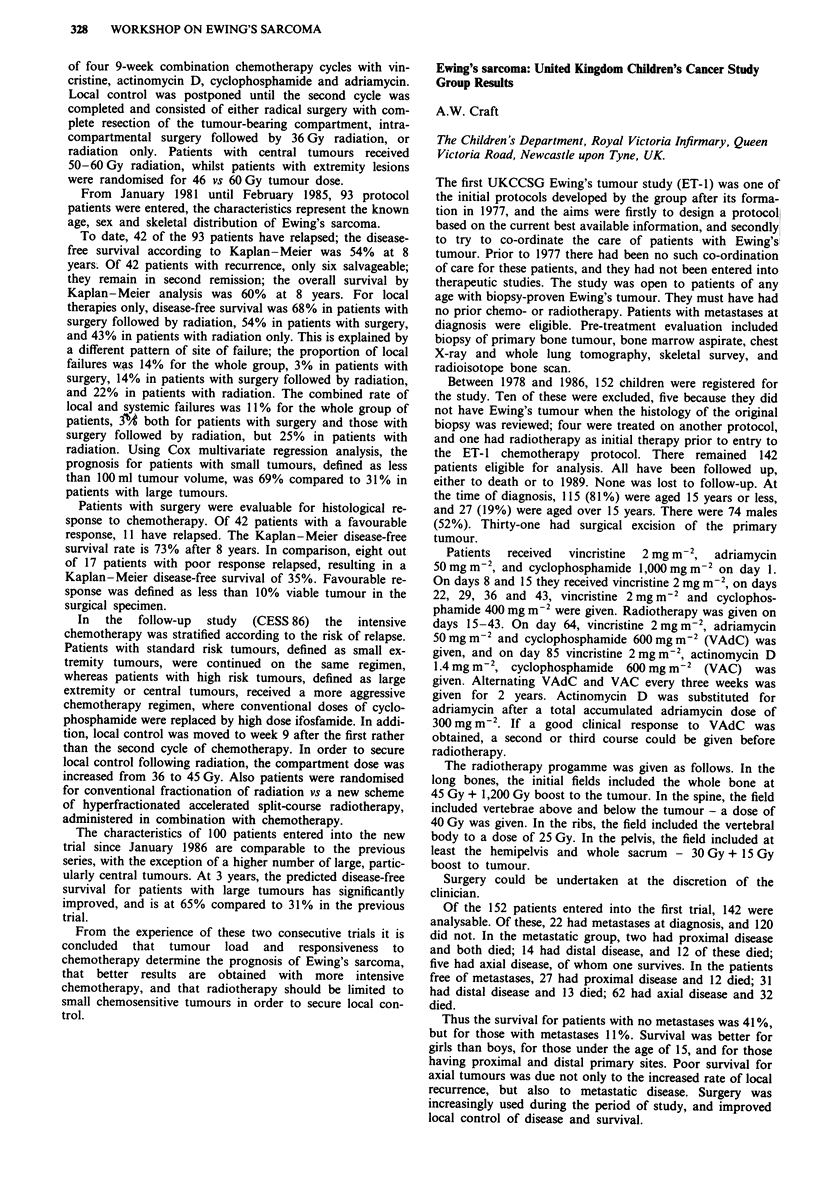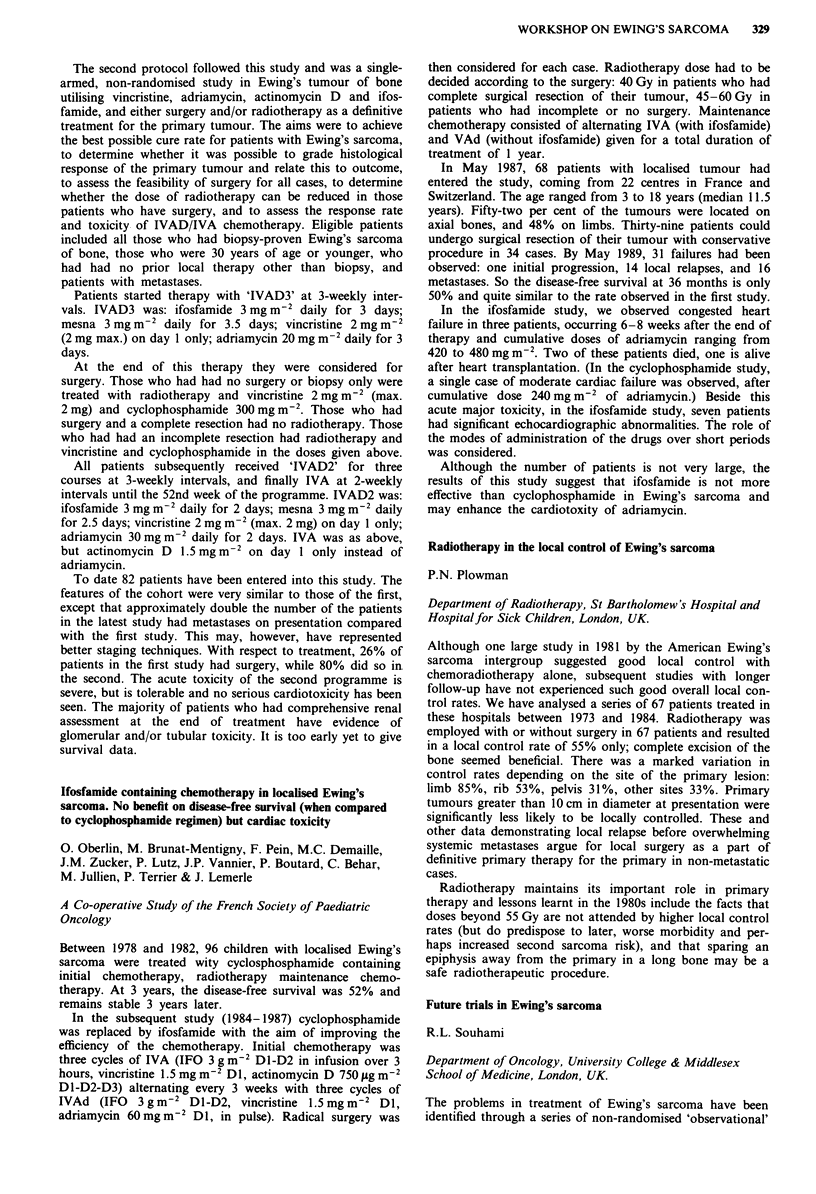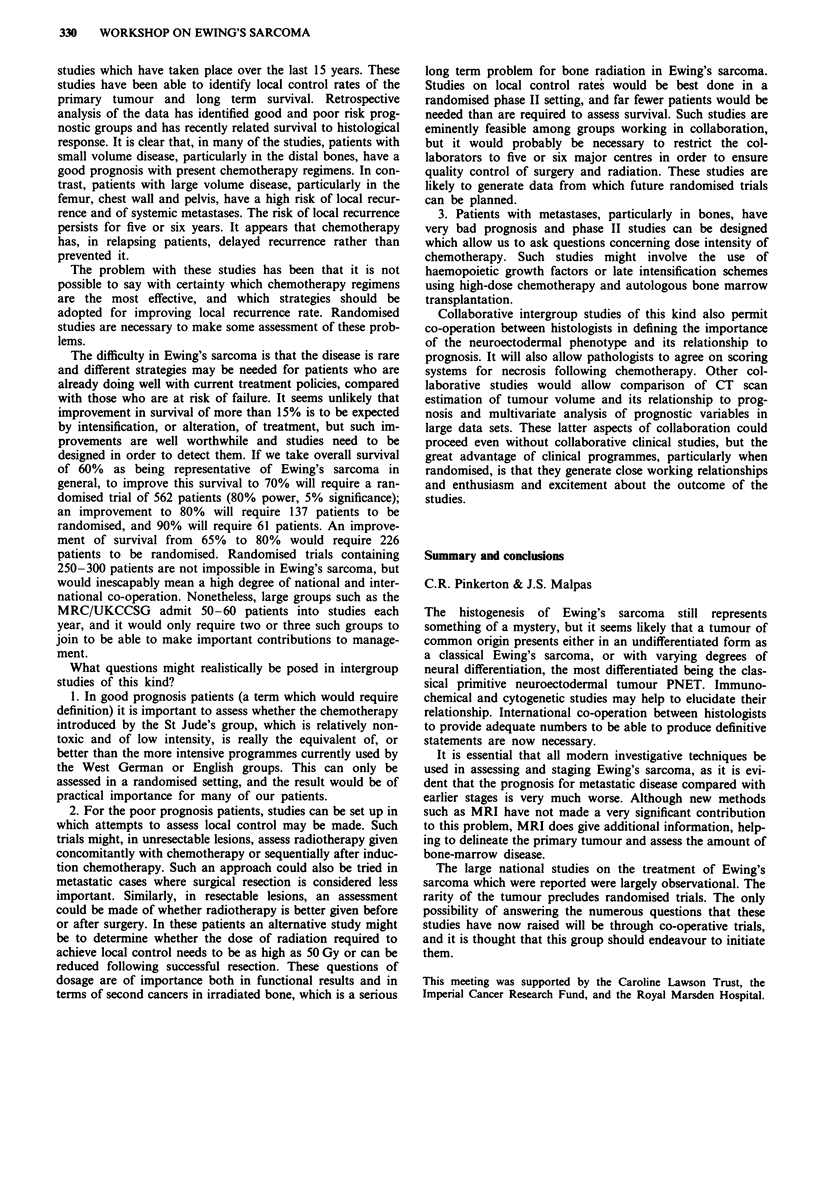# First children's solid tumour group workshop on Ewing's Sarcoma - June 30-July 1, 1989

**Published:** 1990-08

**Authors:** 


Br. J. Cancer (1990), 62, 326-330
© Macmillan Press Ltd., 1990

MEETING REPORT

### First children's solid tumour group workshop on Ewing's sarcoma 30 June to 1 July 1989

*Held at Haslemere, Surrey, UK.*

### Histology of Ewing's sarcoma

A.J. Malcolm

*Department of Pathology, Royal Victoria Infirmary, Newcastle-upon-Tyne, UK.*

Ewing, in 1921, described, a new malignant bone tumour which affected the diaphyses of children and adolescents, which he called a 'malignant endothelioma'. Since then there has been mystery and controversy about whether Ewing's tumour was a distinct entity or merely a useful name for a group of primitive undifferentiated tumours in bone, which included metastases from such tumours as neuroblastoma (a view held by Willis). A malignant tumour composed of glycogen-rich primitive round cells (Ewing's tumour) is now accepted as arising in bone, although the cell of origin remains a speculative issue. The descriptions of Ewing's tumour in the past included cases in which there were poorly-formed Homer-Wright pseudo-rosettes. At present such tumours are regarded as being primitive (peripheral) neuro-ectodermal tumours of bone (PNET). Such tumours react positively with antibodies to neurone specific enolase (NSE) and often with S100 protein, and contain neurofilaments. Electron microscopy shows a few cell processes and occasional neurosecretory granules. This contrasts with the more 'classical' Ewing's tumour which fails to react with any of the 'neural' antibodies and electron microscopy shows cells having a bland primitive appearance containing only a few mitochondria, some lipid and some glycogen. There is no neural differentiation. Between these two extremes there are tumours described as 'atypical' Ewing's tumour which display some reactivity with NSE but not with other 'neural' antibodies. Electron microscopy shows atypical features such as cell processes but there are not neurosecretory granules. The so-called 'Askin' tumour is similar but has some definite neural differentiation. An 11:22 translocation has been reported in examples of each of these tumours. Some typical Ewing's tumours have been reported to develop neural differentiation in tissue culture. This suggests two main possibilities. Either there is an entity 'Ewing's' tumour and a distinctly separate tumour which shows varying neural differentiation called 'PNET' or, alternatively, these tumours are of common origin but show a spectrum of neural differentiation ranging from undifferentiated (Ewing's tumour) to differentiated (PNET). Applying modern immunological techniques retrospectively to 124 cases shows that about 25% of previously described Ewing's tumour have some neural differentiation and might now be called PNETs. However, there was no significant difference in the site, radiological features or clinical outcome between these and Ewing's tumours although all had been treated in an identical manner.

Correspondence: Professor J.S. Malpas, ICRF Department of Medical Oncology, St Bartholomew's Hospital, Little Britain, London EC1A 7BE, UK.
Received 26 October 1989; and in revised form 15 February 1990.

### Gene mapping in relation to the constitutional t(11;22) translocation

B.D. Young, S. Nasipuri & F.E. Cotter

*ICRF Medical Oncology Laboratory, St Bartholomew's Hospital, London, UK.*

Consistent rearrangements to chromosome 11 at bands q23-q24 have been reported in a variety of malignancies. The translocation t(4;11)(q21;q23), first reported by Van den Berghe *et al.* in (1979), is associated with infantile acute lymphoblastic leukaemia while the translocations t(9;11) (p22;q23) and t(11;19)(q23;p13) have been observed in cases of acute monocytic leukaemia. The translocation t(11;22)(q24;q12) is exhibited in 85% of Ewing's sarcoma and is also seen in neuroepithelioma and Askin tumours. Although currently there is little information on the molecular effects of these alterations, it has been established that the *c-ets-1* oncogene on chromosome 11 lies distal to the position of the t(4;11) breakpoint, the t(9;11) breakpoint and proximal to the t(11;22) breakpoint in Ewing's sarcoma. The rare constitutional translocation t(11;22) can be distinguished from the Ewing's sarcoma t(11;22) by the demonstration of *c-ets-1* translocation in the former. A number of genes (including *Thy-1*, *T3δ*, and *c-ets-1*) have been mapped to 11q23 (Human Gene Mapping 9, 1987) but with little information on their relative positions and no conclusive evidence of their activation or alteration in such translocation. It is therefore important to ascertain the positions of these genes relative to the described translocation.

Current methods of gene mapping are relatively time consuming, often involving DNA probe hybridisation to chromosomes *in situ* or to DNA from either somatic cell hybrids or flow-sorted chromosomes. A new approach to gene mapping has been devised which combines enzymatic amplification with high-resolution flow-sorting of human chromosomes. Reliable amplification from as few as 200 chromosomes has been demonstrated. This method, with particular application to mapping the position of chromosomal translocations, has been used to show that the breakpoint for the constitutional translocation t(11;22)(q23;q11) lies proximal to the genes *c-ets-1*, *Thy-1* and *T3δ* and distal to the *int-2* gene. The mapping was confirmed by Southern analysis to much larger numbers of chromosomes sorted from the same cell line. Control reactions for the *bcl-2* gene on chromosome 18 and the C gene of the IGH locus on chromosome 14 demonstrated the discrimination which can be achieved.

The ability to determine the presence or absence of genes or DNA fragments in aliquots of small numbers of chromosomes confers a number of advantages over previous methods of gene mapping. Firstly, the resolution on a cell sorter is often improved by decreasing the flow rate of chromosomes and thus analysis can be performed on smaller fractions of higher purity than previously obtainable for spot-blot hybridisation to flow sorted chromosomes. Secondly the reduced chromosome requirement may open up the possibility of direct studies on chromosomes from tissue previously unsuitable for large scale sorting because of lack of metaphase cells. Studies of Ewing's tumour tissue bearing translocation involving a common region such as chromosome 11q23-q24 may allow further mapping of the region by determining the presence or absence of sequences on the translocated chromosomes. It would be of particular interest to examine directly chromosomes from tumour or leukaemic cells without having to use established cell lines. In this way it may be possible to detect translocations in cells without prior knowledge of karyotype status. Following on from successful amplification from single cells and single sperm cells this approach potentially could be extended to amplification from a single flow-sorted chromosome.

### The role of magnetic resonance imaging (MRI) in Ewing's sarcoma

F.C. Oliff

*Royal Marsden Hospital, Surrey, UK.*

The diagnosis of Ewing's sarcoma is made by a combination of plain film interpretation and histology. The major role of MRI is that of assessing extent of primary tumour because the long scan times required prohibit its use for staging distant metastases and nodal disease. Computed tomography (CT) is the preferred technique for these purposes.

In studies comparing MRI with CT, MRI has been found by some to be better at assessing the extent of marrow disease. The multiplanar imaging of MR allows ready appreciation of the relationship of marrow abnormality to adjacent epiphyseal plate and joint. The soft tissue mass can be identified on T2-weighted images (MR having superior soft tissue contrast to that of CT), and its relationship to the neurovascular bundle is demonstrated without the use of intravenous contrast medium. However, the presence of peritumoral high signal due to oedema can lead to overestimation of tumour extent by as much as 2 cm in the axial plane.

MRI can also be used to evaluate disease following therapy. It does not have any advantage over CT when a simple measurement of tumour size is performed. When signal intensity of tumour on T2-weighted images was evaluated in 60 patients with various bone and soft tissue tumours, no tumour was present in those with low signal (20) (sensitivity 96%) but in those with high signal following surgery (6), tumour was present in all cases. High signal following radiotherapy (34) was due to either inflammation or tumour and differentiation could not be made.

In conclusion, MRI has no role to play in the diagnosis of Ewing's sarcoma, but is superior to plain films and CT for staging the primary tumour. It can delineate the extent of marrow and soft tissue disease but may overestimate tumour bulk due to peritumoral oedema. On follow-up, the absence of high signal on T2-weighted images is reassuring. Following surgery high signal has been shown to be due to tumour, but after radiotherapy, differentiation between tumour and inflammation cannot be made.

### Ewing's sarcoma: the St Jude experience 1978–1988

F.A. Hayes, E.I. Thompson, L. Kun, B. Rao, M. Kumar, W. Myer & H.O. Hustu

*St Jude Children's Research Hospital, Memphis, Tennessee, USA.*

In 1978 a protocol for patients with Ewing's sarcoma (ES) of bone was designed to evaluate (1) the initial response of previously untreated tumour to cyclophosphamide (Cy) and adriamycin (Adr) induction chemotherapy, (2) local control of primary tumours with chemotherapy plus no radiotherapy (RT) (resected lesions) delayed low-dose (30–50 Gy) limited port RT (partial clinical responders) and (3) the overall disease control interval to relatively non-aggressive chemotherapy given for 10–12 months duration.

Eighty patients entered this study, 59 with localised disease and 21 with metastatic tumour. The age range was from 2 years 10 months to 21 years 11 months (median 15 years).

All patients received Cy (150 mg $m^{-2}$ $day^{-1}$ p.o. × 7) and Adr (35 mg $m^{-2}$ on day 8) starting on days 1, 15, 29, 50 and 71. All patients were then evaluated for response to induction therapy. Continuation therapy consisted of vincristine (1.5 mg $m^{-2}$ every week × 11, maximum 2 mg $dose^{-1}$) and dactinomycin (1.5 mg $m^{-2}$ every 2 weeks × 6, maximum 2 mg $dose^{-1}$) followed by a further six cycles of Cy-Adr given at 3-week intervals. Those with metastatic disease then received a further 11 weeks of vincristine and dactinomycin as shown above.

RT was delayed until induction therapy was completed and when delivered was given to limited ports based on post-induction residual lesions. Patients with pulmonary metastases at diagnosis who achieved complete responses with Cy-Adr induction did not receive pulmonary RT. The dose of RT delivered depended on response to induction chemotherapy and surgical resectability of residual lesions.

Of the 21 patients with metastatic tumour, only two failed to achieve a good response to chemotherapy induction. The 5-year disease-free survival (DFS) is 38% and survival (S) is 52% with one relapse occurring beyond 5 years.

Of the 59 patients with localised disease, only four failed to achieve an excellent response to induction chemotherapy. The overall 5-year DFS and S for all 59 patients is 61% and 78% respectively.

The size of the primary lesion at diagnosis was a significant factor in whether or not local control and therefore DFS was maintained. Of 29 patients with lesions less than 8 cm in diameter at diagnosis 27 achieved CR, 21 are disease-free and 25 survive. Eight patients received no RT, 18 low dose RT (three local and two metastatic relapses) and one standard dose RT (one metastatic relapse). Of the 27 achieving CR, three relapsed in the local site and three in a metastatic site for a 5-year DFS of 72% and a S of 83%.

Of the 30 patients with lesions greater than 8 cm at diagnosis, 28 achieved CR, 15 remain free of disease and 20 survive. Six patients received no local RT (two metastatic relapses), 16 received low dose RT (five local, four local plus metastatic relapses), and six received standard dose RT (three local relapses). Of the 28 achieving CR, eight relapsed in the local site, four in the local plus metastatic sites, and two in a metastatic site only for a 5-year DFS of 53% and S of 68%.

The difference between the local control rate in small lesions versus large lesions is significantly different ($P = 0.025$). Local recurrences occurred in the centre of the radiation port in 14 of 15 cases indicating that the small ports of RT delivered did not contribute to the loss of local control.

Results of this study suggest: (1) low doses of Cy-Adr are very effective induction therapy in previously untreated patients with ES; (2) relatively non-aggressive chemotherapy delivered for 10 months results in excellent control of micrometastatic tumour in that only five of 55 responding patients with localised disease relapsed in an isolated metastatic site; (3) in small lesions, therapy as delivered in this study provides disease control as good as or better than that reported with more aggressive therapy, and dose so with minimal toxicity; (4) in larger lesions, therapy as delivered is comparable to other reported studies but as with other studies control is not adequate; (5) markedly limiting the port of RT does not result in 'marginal miss' recurrences and hopefully will decrease long-term growth problems in survivors.

### Ewing's sarcoma in children and adolescents: update of the German GPO-CESS studies

H. Jurgens

*Universitätskinderklinik, Abteilung für Hämatologie und Onkologie, Moorenstr. 5, D-4000 Dusseldorf, FRG.*

Since 1981 the German Society of Paediatric Oncology has conducted two consecutive trials for treatment of primary Ewing's sarcoma of bone. The first trial (CESS 81) consisted of four 9-week combination chemotherapy cycles with vincristine, actinomycin D, cyclophosphamide and adriamycin. Local control was postponed until the second cycle was completed and consisted of either radical surgery with complete resection of the tumour-bearing compartment, intracompartmental surgery followed by 36 Gy radiation, or radiation only. Patients with central tumours received 50–60 Gy radiation, whilst patients with extremity lesions were randomised for 46 *vs* 60 Gy tumour dose.

From January 1981 until February 1985, 93 protocol patients were entered, the characteristics represent the known age, sex and skeletal distribution of Ewing's sarcoma.

To date, 42 of the 93 patients have relapsed; the disease-free survival according to Kaplan–Meier was 54% at 8 years. Of 42 patients with recurrence, only six salvageable; they remain in second remission; the overall survival by Kaplan–Meier analysis was 60% at 8 years. For local therapies only, disease-free survival was 68% in patients with surgery followed by radiation, 54% in patients with surgery, and 43% in patients with radiation only. This is explained by a different pattern of site of failure; the proportion of local failures was 14% for the whole group, 3% in patients with surgery, 14% in patients with surgery followed by radiation, and 22% in patients with radiation. The combined rate of local and systemic failures was 11% for the whole group of patients, 3% both for patients with surgery and those with surgery followed by radiation, but 25% in patients with radiation. Using Cox multivariate regression analysis, the prognosis for patients with small tumours, defined as less than 100 ml tumour volume, was 69% compared to 31% in patients with large tumours.

Patients with surgery were evaluable for histological response to chemotherapy. Of 42 patients with a favourable response, 11 have relapsed. The Kaplan–Meier disease-free survival rate is 73% after 8 years. In comparison, eight out of 17 patients with poor response relapsed, resulting in a Kaplan–Meier disease-free survival of 35%. Favourable response was defined as less than 10% viable tumour in the surgical specimen.

In the follow-up study (CESS 86) the intensive chemotherapy was stratified according to the risk of relapse. Patients with standard risk tumours, defined as small extremity tumours, were continued on the same regimen, whereas patients with high risk tumours, defined as large extremity or central tumours, received a more aggressive chemotherapy regimen, where conventional doses of cyclophosphamide were replaced by high dose ifosfamide. In addition, local control was moved to week 9 after the first rather than the second cycle of chemotherapy. In order to secure local control following radiation, the compartment dose was increased from 36 to 45 Gy. Also patients were randomised for conventional fractionation of radiation *vs* a new scheme of hyperfractionated accelerated split-course radiotherapy, administered in combination with chemotherapy.

The characteristics of 100 patients entered into the new trial since January 1986 are comparable to the previous series, with the exception of a higher number of large, particularly central tumours. At 3 years, the predicted disease-free survival for patients with large tumours has significantly improved, and is at 65% compared to 31% in the previous trial.

From the experience of these two consecutive trials it is concluded that tumour load and responsiveness to chemotherapy determine the prognosis of Ewing's sarcoma, that better results are obtained with more intensive chemotherapy, and that radiotherapy should be limited to small chemosensitive tumours in order to secure local control.

### Ewing's sarcoma: United Kingdom Children's Cancer Study Group Results

A.W. Craft

*The Children's Department, Royal Victoria Infirmary, Queen Victoria Road, Newcastle upon Tyne, UK.*

The first UKCCSG Ewing's tumour study (ET-1) was one of the initial protocols developed by the group after its formation in 1977, and the aims were firstly to design a protocol based on the current best available information, and secondly to try to co-ordinate the care of patients with Ewing's tumour. Prior to 1977 there had been no such co-ordination of care for these patients, and they had not been entered into therapeutic studies. The study was open to patients of any age with biopsy-proven Ewing's tumour. They must have had no prior chemo- or radiotherapy. Patients with metastases at diagnosis were eligible. Pre-treatment evaluation included biopsy of primary bone tumour, bone marrow aspirate, chest X-ray and whole lung tomography, skeletal survey, and radioisotope bone scan.

Between 1978 and 1986, 152 children were registered for the study. Ten of these were excluded, five because they did not have Ewing's tumour when the histology of the original biopsy was reviewed; four were treated on another protocol, and one had radiotherapy as initial therapy prior to entry to the ET-1 chemotherapy protocol. There remained 142 patients eligible for analysis. All have been followed up, either to death or to 1989. None was lost to follow-up. At the time of diagnosis, 115 (81%) were aged 15 years or less, and 27 (19%) were aged over 15 years. There were 74 males (52%). Thirty-one had surgical excision of the primary tumour.

Patients received vincristine 2 mg $m^{-2}$, adriamycin 50 mg $m^{-2}$, and cyclophosphamide 1,000 mg $m^{-2}$ on day 1. On days 8 and 15 they received vincristine 2 mg $m^{-2}$, on days 22, 29, 36 and 43, vincristine 2 mg $m^{-2}$ and cyclophosphamide 400 mg $m^{-2}$ were given. Radiotherapy was given on days 15–43. On day 64, vincristine 2 mg $m^{-2}$, adriamycin 50 mg $m^{-2}$ and cyclophosphamide 600 mg $m^{-2}$ (VAdC) was given, and on day 85 vincristine 2 mg $m^{-2}$, actinomycin D 1.4 mg $m^{-2}$, cyclophosphamide 600 mg $m^{-2}$ (VAC) was given. Alternating VAdC and VAC every three weeks was given for 2 years. Actinomycin D was substituted for adriamycin after a total accumulated adriamycin dose of 300 mg $m^{-2}$. If a good clinical response to VAdC was obtained, a second or third course could be given before radiotherapy.

The radiotherapy progamme was given as follows. In the long bones, the initial fields included the whole bone at 45 Gy + 1,200 Gy boost to the tumour. In the spine, the field included vertebrae above and below the tumour – a dose of 40 Gy was given. In the ribs, the field included the vertebral body to a dose of 25 Gy. In the pelvis, the field included at least the hemipelvis and whole sacrum – 30 Gy + 15 Gy boost to tumour.

Surgery could be undertaken at the discretion of the clinician.

Of the 152 patients entered into the first trial, 142 were analysable. Of these, 22 had metastases at diagnosis, and 120 did not. In the metastatic group, two had proximal disease and both died; 14 had distal disease, and 12 of these died; five had axial disease, of whom one survives. In the patients free of metastases, 27 had proximal disease and 12 died; 31 had distal disease and 13 died; 62 had axial disease and 32 died.

Thus the survival for patients with no metastases was 41%, but for those with metastases 11%. Survival was better for girls than boys, for those under the age of 15, and for those having proximal and distal primary sites. Poor survival for axial tumours was due not only to the increased rate of local recurrence, but also to metastatic disease. Surgery was increasingly used during the period of study, and improved local control of disease and survival.

The second protocol followed this study and was a single-armed, non-randomised study in Ewing's tumour of bone utilising vincristine, adriamycin, actinomycin D and ifosfamide, and either surgery and/or radiotherapy as a definitive treatment for the primary tumour. The aims were to achieve the best possible cure rate for patients with Ewing's sarcoma, to determine whether it was possible to grade histological response of the primary tumour and relate this to outcome, to assess the feasibility of surgery for all cases, to determine whether the dose of radiotherapy can be reduced in those patients who have surgery, and to assess the response rate and toxicity of IVAD/IVA chemotherapy. Eligible patients included all those who had biopsy-proven Ewing's sarcoma of bone, those who were 30 years of age or younger, who had had no prior local therapy other than biopsy, and patients with metastases.

Patients started therapy with 'IVAD3' at 3-weekly intervals. IVAD3 was: ifosfamide 3 mg $m^{-2}$ daily for 3 days; mesna 3 mg $m^{-2}$ daily for 3.5 days; vincristine 2 mg $m^{-2}$ (2 mg max.) on day 1 only; adriamycin 20 mg $m^{-2}$ daily for 3 days.

At the end of this therapy they were considered for surgery. Those who had had no surgery or biopsy only were treated with radiotherapy and vincristine 2 mg $m^{-2}$ (max. 2 mg) and cyclophosphamide 300 mg $m^{-2}$. Those who had surgery and a complete resection had no radiotherapy. Those who had had an incomplete resection had radiotherapy and vincristine and cyclophosphamide in the doses given above.

All patients subsequently received 'IVAD2' for three courses at 3-weekly intervals, and finally IVA at 2-weekly intervals until the 52nd week of the programme. IVAD2 was: ifosfamide 3 mg $m^{-2}$ daily for 2 days; mesna 3 mg $m^{-2}$ daily for 2.5 days; vincristine 2 mg $m^{-2}$ (max. 2 mg) on day 1 only; adriamycin 30 mg $m^{-2}$ daily for 2 days. IVA was as above, but actinomycin D 1.5 mg $m^{-2}$ on day 1 only instead of adriamycin.

To date 82 patients have been entered into this study. The features of the cohort were very similar to those of the first, except that approximately double the number of the patients in the latest study had metastases on presentation compared with the first study. This may, however, have represented better staging techniques. With respect to treatment, 26% of patients in the first study had surgery, while 80% did so in the second. The acute toxicity of the second programme is severe, but is tolerable and no serious cardiotoxicity has been seen. The majority of patients who had comprehensive renal assessment at the end of treatment have evidence of glomerular and/or tubular toxicity. It is too early yet to give survival data.

**Ifosfamide containing chemotherapy in localised Ewing's sarcoma. No benefit on disease-free survival (when compared to cyclophosphamide regimen) but cardiac toxicity**

O. Oberlin, M. Brunat-Mentigny, F. Pein, M.C. Demaille, J.M. Zucker, P. Lutz, J.P. Vannier, P. Boutard, C. Behar, M. Jullien, P. Terrier & J. Lemerle

*A Co-operative Study of the French Society of Paediatric Oncology*

Between 1978 and 1982, 96 children with localised Ewing's sarcoma were treated wity cyclosphosphamide containing initial chemotherapy, radiotherapy maintenance chemotherapy. At 3 years, the disease-free survival was 52% and remains stable 3 years later.

In the subsequent study (1984–1987) cyclophosphamide was replaced by ifosfamide with the aim of improving the efficiency of the chemotherapy. Initial chemotherapy was three cycles of IVA (IFO 3 g $m^{-2}$ D1-D2 in infusion over 3 hours, vincristine 1.5 mg $m^{-2}$ D1, actinomycin D 750 $\mu$g $m^{-2}$ D1-D2-D3) alternating every 3 weeks with three cycles of IVAd (IFO 3 g $m^{-2}$ D1-D2, vincristine 1.5 mg $m^{-2}$ D1, adriamycin 60 mg $m^{-2}$ D1, in pulse). Radical surgery was then considered for each case. Radiotherapy dose had to be decided according to the surgery: 40 Gy in patients who had complete surgical resection of their tumour, 45–60 Gy in patients who had incomplete or no surgery. Maintenance chemotherapy consisted of alternating IVA (with ifosfamide) and VAd (without ifosfamide) given for a total duration of treatment of 1 year.

In May 1987, 68 patients with localised tumour had entered the study, coming from 22 centres in France and Switzerland. The age ranged from 3 to 18 years (median 11.5 years). Fifty-two per cent of the tumours were located on axial bones, and 48% on limbs. Thirty-nine patients could undergo surgical resection of their tumour with conservative procedure in 34 cases. By May 1989, 31 failures had been observed: one initial progression, 14 local relapses, and 16 metastases. So the disease-free survival at 36 months is only 50% and quite similar to the rate observed in the first study.

In the ifosfamide study, we observed congested heart failure in three patients, occurring 6–8 weeks after the end of therapy and cumulative doses of adriamycin ranging from 420 to 480 mg $m^{-2}$. Two of these patients died, one is alive after heart transplantation. (In the cyclophosphamide study, a single case of moderate cardiac failure was observed, after cumulative dose 240 mg $m^{-2}$ of adriamycin.) Beside this acute major toxicity, in the ifosfamide study, seven patients had significant echocardiographic abnormalities. The role of the modes of administration of the drugs over short periods was considered.

Although the number of patients is not very large, the results of this study suggest that ifosfamide is not more effective than cyclophosphamide in Ewing's sarcoma and may enhance the cardiotoxity of adriamycin.

**Radiotherapy in the local control of Ewing's sarcoma**

P.N. Plowman

*Department of Radiotherapy, St Bartholomew's Hospital and Hospital for Sick Children, London, UK.*

Although one large study in 1981 by the American Ewing's sarcoma intergroup suggested good local control with chemoradiotherapy alone, subsequent studies with longer follow-up have not experienced such good overall local control rates. We have analysed a series of 67 patients treated in these hospitals between 1973 and 1984. Radiotherapy was employed with or without surgery in 67 patients and resulted in a local control rate of 55% only; complete excision of the bone seemed beneficial. There was a marked variation in control rates depending on the site of the primary lesion: limb 85%, rib 53%, pelvis 31%, other sites 33%. Primary tumours greater than 10 cm in diameter at presentation were significantly less likely to be locally controlled. These and other data demonstrating local relapse before overwhelming systemic metastases argue for local surgery as a part of definitive primary therapy for the primary in non-metastatic cases.

Radiotherapy maintains its important role in primary therapy and lessons learnt in the 1980s include the facts that doses beyond 55 Gy are not attended by higher local control rates (but do predispose to later, worse morbidity and perhaps increased second sarcoma risk), and that sparing an epiphysis away from the primary in a long bone may be a safe radiotherapeutic procedure.

**Future trials in Ewing's sarcoma**

R.L. Souhami

*Department of Oncology, University College & Middlesex School of Medicine, London, UK.*

The problems in treatment of Ewing's sarcoma have been identified through a series of non-randomised 'observational'

studies which have taken place over the last 15 years. These studies have been able to identify local control rates of the primary tumour and long term survival. Retrospective analysis of the data has identified good and poor risk prognostic groups and has recently related survival to histological response. It is clear that, in many of the studies, patients with small volume disease, particularly in the distal bones, have a good prognosis with present chemotherapy regimens. In contrast, patients with large volume disease, particularly in the femur, chest wall and pelvis, have a high risk of local recurrence and of systemic metastases. The risk of local recurrence persists for five or six years. It appears that chemotherapy has, in relapsing patients, delayed recurrence rather than prevented it.

The problem with these studies has been that it is not possible to say with certainty which chemotherapy regimens are the most effective, and which strategies should be adopted for improving local recurrence rate. Randomised studies are necessary to make some assessment of these problems.

The difficulty in Ewing's sarcoma is that the disease is rare and different strategies may be needed for patients who are already doing well with current treatment policies, compared with those who are at risk of failure. It seems unlikely that improvement in survival of more than 15% is to be expected by intensification, or alteration, of treatment, but such improvements are well worthwhile and studies need to be designed in order to detect them. If we take overall survival of 60% as being representative of Ewing's sarcoma in general, to improve this survival to 70% will require a randomised trial of 562 patients (80% power, 5% significance); an improvement to 80% will require 137 patients to be randomised, and 90% will require 61 patients. An improvement of survival from 65% to 80% would require 226 patients to be randomised. Randomised trials containing 250–300 patients are not impossible in Ewing's sarcoma, but would inescapably mean a high degree of national and international co-operation. Nonetheless, large groups such as the MRC/UKCCSG admit 50–60 patients into studies each year, and it would only require two or three such groups to join to be able to make important contributions to management.

What questions might realistically be posed in intergroup studies of this kind?

1. In good prognosis patients (a term which would require definition) it is important to assess whether the chemotherapy introduced by the St Jude's group, which is relatively non-toxic and of low intensity, is really the equivalent of, or better than the more intensive programmes currently used by the West German or English groups. This can only be assessed in a randomised setting, and the result would be of practical importance for many of our patients.

2. For the poor prognosis patients, studies can be set up in which attempts to assess local control may be made. Such trials might, in unresectable lesions, assess radiotherapy given concomitantly with chemotherapy or sequentially after induction chemotherapy. Such an approach could also be tried in metastatic cases where surgical resection is considered less important. Similarly, in resectable lesions, an assessment could be made of whether radiotherapy is better given before or after surgery. In these patients an alternative study might be to determine whether the dose of radiation required to achieve local control needs to be as high as 50 Gy or can be reduced following successful resection. These questions of dosage are of importance both in functional results and in terms of second cancers in irradiated bone, which is a serious long term problem for bone radiation in Ewing's sarcoma. Studies on local control rates would be best done in a randomised phase II setting, and far fewer patients would be needed than are required to assess survival. Such studies are eminently feasible among groups working in collaboration, but it would probably be necessary to restrict the collaborators to five or six major centres in order to ensure quality control of surgery and radiation. These studies are likely to generate data from which future randomised trials can be planned.

3. Patients with metastases, particularly in bones, have very bad prognosis and phase II studies can be designed which allow us to ask questions concerning dose intensity of chemotherapy. Such studies might involve the use of haemopoietic growth factors or late intensification schemes using high-dose chemotherapy and autologous bone marrow transplantation.

Collaborative intergroup studies of this kind also permit co-operation between histologists in defining the importance of the neuroectodermal phenotype and its relationship to prognosis. It will also allow pathologists to agree on scoring systems for necrosis following chemotherapy. Other collaborative studies would allow comparison of CT scan estimation of tumour volume and its relationship to prognosis and multivariate analysis of prognostic variables in large data sets. These latter aspects of collaboration could proceed even without collaborative clinical studies, but the great advantage of clinical programmes, particularly when randomised, is that they generate close working relationships and enthusiasm and excitement about the outcome of the studies.

### Summary and conclusions

C.R. Pinkerton & J.S. Malpas

The histogenesis of Ewing's sarcoma still represents something of a mystery, but it seems likely that a tumour of common origin presents either in an undifferentiated form as a classical Ewing's sarcoma, or with varying degrees of neural differentiation, the most differentiated being the classical primitive neuroectodermal tumour PNET. Immunochemical and cytogenetic studies may help to elucidate their relationship. International co-operation between histologists to provide adequate numbers to be able to produce definitive statements are now necessary.

It is essential that all modern investigative techniques be used in assessing and staging Ewing's sarcoma, as it is evident that the prognosis for metastatic disease compared with earlier stages is very much worse. Although new methods such as MRI have not made a very significant contribution to this problem, MRI does give additional information, helping to delineate the primary tumour and assess the amount of bone-marrow disease.

The large national studies on the treatment of Ewing's sarcoma which were reported were largely observational. The rarity of the tumour precludes randomised trials. The only possibility of answering the numerous questions that these studies have now raised will be through co-operative trials, and it is thought that this group should endeavour to initiate them.

This meeting was supported by the Caroline Lawson Trust, the Imperial Cancer Research Fund, and the Royal Marsden Hospital.